# Mechanical and Morphological Properties of Waterborne ABA Hard-Soft-Hard Block Copolymers Synthesized by Means of RAFT Miniemulsion Polymerization

**DOI:** 10.3390/polym11081259

**Published:** 2019-07-30

**Authors:** Gordana Siljanovska Petreska, Arantxa Arbe, Clemens Auschra, Maria Paulis

**Affiliations:** 1 POLYMAT, University of the Basque Country UPV/EHU, Joxe Mari Korta Center, Avda. Tolosa 72, 20018 Donostia-San Sebastián, Spain; 2 BASF SE, 67056 Ludwigshafen, Germany; 3 Centro de Física de Materiales (CFM) (CSIC–UPV/EHU)—Materials Physics Center (MPC), Paseo Manuel de Lardizabal 5, 20018 Donostia-San Sebastián, Spain

**Keywords:** RAFT, miniemulsion polymerization, ABA hard-soft-hard block copolymers, particle and film morphology, films viscoelastic properties, temperature responsiveness

## Abstract

High molecular weight waterborne ABA block copolymers of styrene (St) and 2-ethylhexyl acrylate (2EHA) containing hard and soft domains were synthesized by means of RAFT (mini)emulsion polymerization using a bifunctional symmetric *S,S*-dibenzyl trithiocarbonate (DBTTC) RAFT agent. Miniemulsion polymerization was initially used for the synthesis of the A-block, which forms hard domains, followed by 2EHA pre-emulsion feeding to build the B-block soft domains. Polymerization kinetics and the evolution of the Molecular Weight Distribution (MWD) were followed during the synthesis of different ABA block copolymers. The thermal properties of the final symmetric block copolymers were studied on dried films by means of DSC. It was found that the block copolymers have two glass transitions, which indicates the presence of a two-phase system. Phase separation was investigated by means of microscopic techniques (AFM and TEM) and SAXS, both of the particles in the latex form, as well as after film formation at room temperature and after different post-treatments. Films were annealed at temperatures well above the glass transition temperature (*T*_g_) of the hard phase to study the bulk morphology of the films after complete particle coalescence. Moreover, for comparison purposes, the films were re-dissolved in THF, and films were again cast directly from the homogeneous THF solutions. As THF is a good solvent for both blocks, such films serve as a reference for the equilibrium morphology. Finally, DMTA studies of the films annealed at different temperatures were performed to correlate the morphology changes with the mechanical properties of the block copolymers.

## 1. Introduction

Well-defined block copolymers preparation is not only a synthetic challenge, but it is also of high interest for the design of novel polymeric materials that provide superior combinations of properties. In this context, controlled radical polymerization, including the three different methods (nitroxide-mediated radical polymerization (NMP) [1], atom transfer radical polymerization (ATRP) [2] and reversible addition fragmentation chain-transfer polymerization (RAFT) [3]), has in the past years emerged as a technique of choice to prepare block, graft or comb copolymers of precise molecular and chemical structure. These types of polymers are increasingly involved in traditional structural materials [4] as well as in higher added value applications such as core-shell functional nanoparticles [5], organic/inorganic nanoparticles [6] or block copolymer lithography [7,8]. For the development of materials with unique combinations of properties, the self-assembling capability of block copolymers and the resulting regular nanostructures are indeed highly appreciated.

Block copolymers composed of different incompatible polymer segments connected by covalent bonds spontaneously form microphase-separated structures ranging from several nanometers to 100 nm in length. In general, the self-assembly of bulk block copolymers into phase-separated structures (e.g., spherical, cylindrical, lamellar and bicontinuous structures) is governed by both the enthalpic interaction between the blocks, quantified by the Flory-Huggins interaction parameter χ, and by the total degree of polymerization of the copolymer, N. Generally, if the product of χ and N exceeds 10.5, then the block copolymers phase separate into domains whose morphology depends on the relative volume fractions of the blocks [9].

Emulsion polymerization is a process of significant importance for industrial production because it is simple, economic and in addition solvent-free and is thus very attractive from an environmental point of view. From an application perspective, block copolymers directly obtained as a stable latex form should produce useful nanostructured films, which can be applied as compatibilizers or as nanostructuring agents to be blended with homopolymers [4]. Polymerization in a water dispersed system offers the possibility to obtain polymer nanostructures directly inside the latex particle, which is not a possibility offered by conventional solution or bulk block copolymers synthesis. To obtain such nanostructured polymer particles dispersed in water, several approaches can be found in literature. For instance, Okubo et al. reported two main approaches to prepare nanostructured polymer particles [6,10,11,12]. In the first approach, multilayered polystyrene/poly(methyl methacrylate) composite microparticles were prepared by the so-called solvent-absorbing/releasing method [6,10,11], using either graft copolymers (polystyrene-*g*-polymethyl methacrylate) or block copolymers (polystyrene-*b*-polymethyl methacrylate) as compatibilizers. In the second approach, they used two-step ATRP miniemulsion polymerization process to prepare poly(*i*-butyl methacrylate)-*b*-polystyrene nanostructured latex particles [12]. Later on, Nicolas et al. [13] produced stable latexes with up to 27 wt % solids content of diblock and triblock copolymers by NMP, comprising a poly(*n*-butyl acrylate) first/central block and polystyrene second/outer block. For this purpose, they used (mini)emulsion polymerization, and monofunctional or difunctional water-soluble alkoxyamine initiators. They studied the direct self-assembly of block copolymers within latex particles in films dried at room temperature, the morphological changes of the films after annealing and in solvent cast films. Thermal properties of the films were studied using differential scanning calorimetry (DSC). However, no particle morphology nor mechanical properties of the films were provided. 

Water dispersed particles containing block copolymers of AB [14,15,16] and ABA type [14] have also been synthesized using RAFT miniemulsion polymerization. Nevertheless, the authors scarcely explored the mechanical and morphological properties of the polymer latexes and the films cast at different conditions. Nanostructured particles made of styrene (St) and butadiene (Bd) prepared by RAFT polymerization were reported by Wei et al. [17]. They studied the latex morphology and showed that the morphology is changing with the increase of Bd segment. However, the authors did not provide any film morphology nor mechanical properties in the article. Binary monomers RAFT miniemulsion copolymerization kinetic modelling was developed by Xiong et al. [18] to predict and produce well-defined p(St-*b*-St/Bd) copolymers. The morphology of the films cast from toluene solution was analyzed by the authors and different morphologies were shown. Nevertheless, the author did not provide the particle morphology nor the morphology of the films casted directly from the latex. Gel-free styrene-butadiene-styrene triblock copolymers latex were synthesized by Wei et al. [19] and the authors only showed the morphology and mechanical properties of the polymer films cast from THF solutions. Highly symmetric poly(St)-block-poly(Bd-stat-St)-block-poly(St) copolymers were synthesized in a non-stop one-pot RAFT polymerization in miniemulsion by Landfester et al. [20] with long polymerization times. The synthesized polymer particles showed core-shell morphology and the shell was turning bilayer as the Bd content increased. Nevertheless, the authors did not provide the equilibrium morphology of the obtained block copolymers. ABA triblock copolymers were also synthesized by Yang and et al. [21] by means of RAFT miniemulsion polymerization by initially preparing the macroRAFT in solution polymerization. The authors studied the particles and film morphologies and concluded that thermal annealing of the film did not attain the thermodynamic equilibrium. 

The approach proposed in this work goes a step further, as it aims at preparing symmetric block copolymers by means of waterborne dispersed system (without the use of any solvent in any step of the process), at moderate solids content, analyzing the phase separation obtained in the particles and in the films and correlating that morphology with the final mechanical properties of the films prepared at different temperatures. Therefore, their possible use in temperature responsive materials can be envisaged. We have previously synthesized ABA crystalline-soft-crystalline block copolymers by RAFT miniemulsion polymerization and carried out a similar study [22], but the modulus of the annealed films was found to be lower than that of the non annealed films. In order to search for a higher modulus in the annealed films, the semi-crystalline moiety has been replaced by a hard moiety in this work. Therefore, in this article ABA triblock copolymers have been synthesized by means of RAFT miniemulsion polymerization with A blocks composed of hard polystyrene and B block of soft poly2EHA domains. The effect that confined phase separated particle morphology has on film formation process was studied. Furthermore, the effect of different film formation conditions on the thermoresponsive mechanical properties of the final films was analyzed.

## 2. Materials and Methods

### 2.1. Materials

For the synthesis of the block copolymers technical grade styrene (St, 99.7% purity, 10–20 ppm inhibitor 4-tert-butylcatechol) and 2-ethylhexyl acrylate (2EHA, 10–20 ppm inhibitor (4-methoxyphenol)), monomers from Quimidroga (Barcelona, Spain) were used. Stearyl acrylate (SA, Sigma Aldrich, Madrid, Spain) was added as a co-stabilizer to prevent Ostwald ripening and sodium bicarbonate (NaHCO_3,_ Aldrich) was added as a buffer to control the miniemulsion viscosity by reducing the electrostatic interactions among droplets. To stabilize the droplets, alkyldiphenyloxide disulfonate -Dowfax 2A1 (45 wt % active content, DOW Chemicals, Madrid, Spain) was used as an anionic surfactant and Disponil A3065 (65 wt % active content, BASF Company, Ludwigshafen, Germany) as a non-ionic surfactant. To initiate the polymerization the oil soluble thermal initiator, azobisisobutyronitrile (AIBN, purity 98%, Sigma Aldrich) was added. *S,S*-Dibenzyl trithiocarbonate (DBTTC, kindly supplied by Arkema) was used as a bifunctional symmetric RAFT agent to mediate the polymerization. All the chemicals were used as received without any further purification. Deionized MilliQ water was used as polymerization media and 1 wt % hydroquinone (HQ, purity 99%, Sigma Aldrich, Madrid, Spain) water solution was used for quenching the reaction in the samples withdrawn from the reactor at certain time intervals. Tetrahydrofuran (THF, 99.9% GPC, Scharlab, Barcelona, Spain) and toluene (Sigma Aldrich) were used as solvents for the GPC analysis.

### 2.2. Methods

#### 2.2.1. Synthesis of the First A Block: Batch Miniemulsion Polymerization

Polystyrene (pSt) “A” hard block was synthesized via RAFT miniemulsion polymerization at 30% solids content (s.c.) in water. The miniemulsion was prepared according to the following procedure and optimized recipe, which was obtained after performing preliminary reactions. Initially, the water phase was prepared by dissolving the surfactants Dowfax 2A (2 wt % active surfactant based on styrene monomer, BSM) and Disponil A3065 (1 wt % active surfactant BSM) and NaHCO_3_ (0.16 wt % BSM) in deionized water. The ingredients were mixed in a beaker using a magnetic stirrer for several minutes. The oil phase was prepared by dissolving the stearyl acrylate costabilizer (SA, 8 wt % BSM), RAFT agent (DBTTC) and initiator (AIBN) in styrene. Then, the organic phase was added to the water phase and the coarse emulsion obtained after agitation was then ultrasonicated for 15 min using Branson Digital Sonifier 450 (amplitude 70% and 50% duty cycle, Danbury, Connecticut, USA) under magnetic stirring in an ice-water bath. Once the miniemulsion was prepared, it was transferred to a jacketed batch reactor and was purged with nitrogen for 30 min under agitation (60 rpm). Then, heating started and when the desired polymerization temperature was reached, the agitation was increased to 100 rpm and time 0 was marked. The reaction was performed for 360 min. The molar ratio of (RAFT):(Initiator) used was 5:1.

#### 2.2.2. Synthesis of the Second Block: Semi-batch Emulsion Polymerization

Polystyrene initial A block served as a seed for the synthesis of the second middle B block. ABA block copolymers were synthesized at a total solids content of 30%. The B block was formed by feeding the monomer (2EHA) as a pre-emulsion for 3 h and continuing the polymerization for 2 h more, batchwise. The pre-emulsion consisted of monomer, water and the emulsifiers (Dowfax 2A and Disponil A3065). The total amount of surfactants was 3 wt % based on all monomers. To start the polymerization, an additional amount of AIBN dissolved in monomer (1.76–3.54 wt % in 2EHA) was added once the temperature reached 70 °C. The molar ratio of (RAFT):(Initiator) was 2:1. The seed, the pre-emulsion and the initiator solution were purged with nitrogen for 30 min prior to polymerization and a slow flow of nitrogen was maintained during the whole polymerization. For all block copolymer latexes, the coagulum content was found to be lower than 1 wt %.

Stability of the miniemulsion: the stability of the miniemulsions was determined by means of a Turbiscan LabExpert apparatus (Formulaction, Toulouse, France). In this equipment, the miniemulsion was placed in a vial (55 mm path length) heated at 60 °C for 6 h and the stability was determined by studying the evolution of the light backscattered by the miniemulsions along the vial height.

Conversion measurements: the monomers conversion was followed gravimetrically. The samples were withdrawn from the reactor, put in an aluminum cup and a drop of 1 wt % of hydroquinone was added. Then, the cup was placed in an oven at 60 °C overnight. The overall conversion was calculated from the measured dry weight residue.

Monomer droplet and particle sizes were measured using dynamic light scattering (Zetasizer Nano Z, Malvern Instruments, Malvern, UK). A drop of either the miniemulsion or latex was diluted in distilled water by a factor of 100 and then measured. Reported droplets and particle diameters are a mean of the Z-average of three measurements, each of them analyzed by 11 runs of 30 s each.

Molecular Weight Distribution (MWD) and the average molecular weight of the obtained latexes were determined by size exclusion chromatography (SEC-GPC) at 35 °C using solvent delivery unit LC-20AD (Shimadzu Corp., Kyoto, JApan The setting consisted of a pump, a refractive index detector (Waters Corp. 2410) and three columns in series (Styragel HR2, HR4 and HR6; with pore sizes from 10^2^ to 10^6^ Å). THF was used as solvent at a flow rate of 1 mL/min. The polymer obtained after drying the latex sample in an oven overnight was dissolved into THF and filtered (polyamide filter Φ = 0.45 μm) before the analysis. Then, toluene (100 μL) was added as internal standard prior injection in the SEC-GPC. 18 polystyrene standards with narrow MWD and with *M*_n_ ranging from 555 to 3,690,000 g/mol (Polymer Laboratories) were used for the calibration of the equipment.

Thermal characterization. The glass transition temperature of the room temperature cast films was measured on a DSC, Q1000, TA Instruments (Hüllhorst, Germany). The scanning cycles consisted of first cooling to −80 °C at 10 °C·min^−1^ (isothermal for 2 min), then heating to 150 °C (isothermal for 2 min), second cooling to −80 °C (isothermal for 2 min) at 10 °C min^−1^ and then heating to 150 °C at 10 °C·min^−1^ and cooling again to 25 °C. The second heating cycle was used for the determination of the *T*_g_.

Thermal stability. ABA block copolymers thermal stability was measured using thermal Gravimetric Analysis (TGA) on a TGA Q 500 (TA, Hüllhorst, Germany) instrument at a heating rate of 10 °C·min^−1^ from 40 to 800 °C in nitrogen atmosphere.

Dynamic Mechanical Thermal analysis (DMTA) measurements were performed in tensile geometry on 0.6–0.9 mm thick films using Q800 von TA-Instruments, a heating rate 4 °C min^−1^, frequency of 1 Hz and constant strain.

The morphology of the samples at different conditions was investigated using both transition electron microscopy (TEM), atomic force microscopy (AFM) and small angle X-ray scattering (SAXS). See Supplementary Material for the detailed description of the procedures followed with these techniques.

The quantification of each phase in the block copolymers, i.e., the quantification of the monomer composition, was determined by NMR (see Supplementary Material). ^1^H NMR spectra were recorded on a Bruker 400 AVANCE equipped with a z gradient BBO probe using CDCl_3_ as solvent.

## 3. Results and Discussion

### 3.1. RAFT Mediated Miniemulsion Polymerization

**Figure 1:**
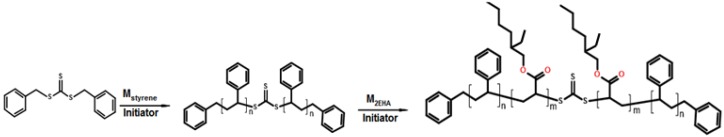
Scheme of p(St-2HA-St) block copolymer formation.

DBTTC is a symmetrical RAFT agent that has two homolytic leaving R groups, which enable the polymerization of ABA triblock copolymers in two sequential monomer additional steps as shown in Figure 1.

When the initial monomer is added it inserts in between the leaving groups and –S(C=S)- moiety. The first polystyrene (A) block is grown this way and is extended by the second monomer addition (2EHA, B block), which accommodates in the middle and shifts the A block to the outside of the polymer chain.

Polystyrene A blocks with targeted *M*_n_ of 30,000 (pSt30) and 50,000 g/mol (pSt50) were synthesized using the following equation:

(1) $$\bar{Mn_{theory}}=M_{RAFT}+\frac{x\left[M\right]oM_{M}}{\left[RAFT\right]o},$$

where $\bar{Mn_{theory}}$ represents the predicted molecular weight, $M_{M}$ represent the molecular weight of the monomer, $M_{RAFT}$ is the molecular weight of the RAFT agent, x is the conversion and [M]o and [RAFT]o are the initial moles of the monomer and the RAFT agent, respectively. The amount of initiator was not considered in the calculation of the theoretical *M*_n_ due to the relatively small amount used compared to the RAFT agent. This approach was also used by de Brouwer [23], who disregards the chains derived from the initiator in the calculation, due to the low efficiency of initiator systems such as AIBN in heterogeneous polymerizations. The amount of co-stabilizer used was also not included in the calculation of the targeted *M*_n_. SA is an extremely hydrophobic monomer that was used to prevent the Oswald ripening in the miniemulsions [24,25]. It incorporates in the polymer backbone upon polymerization and stays in the film upon film formation, unlike the widely used hexadecane co-stabilizer, which evaporates into the atmosphere and increases the volatile organic content of the latex.

**Figure 2:**
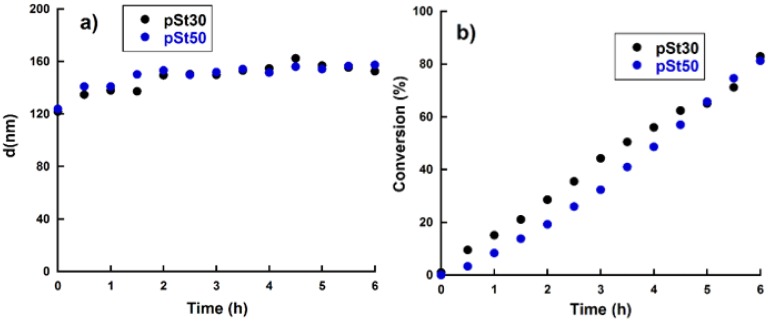
Evolution of (**a**) droplet/particle size evolution with time; (**b**) St conversion versus time for the miniemulsion polymerizations carried out to obtain pSt30 and pSt50.

Prior to polymerization, the stability of the miniemulsion was investigated by Turbiscan measurements and the results showed excellent stability during 6 h at 60 °C (see Figure S2 in Supporting Material). Polystyrene initial blocks were then synthesized using a molar ratio of RAFT:AIBN = 5:1, at a reaction temperature of 70 °C and with a reaction time of 6 h. The evolution of the droplet-particle size is shown in Figure 2a. It can be seen from the graph that dp initially increases in the first 2 h with the progress of the Ostwald ripening process, and ultimately a relatively stationary particle size is achieved. The particle size at the end of the reactions was 30 nm higher than the droplet size for both pSt30 and pSt50.

The conversion versus time plot is shown in Figure 2b and one can observe that the conversion increment with time was not linear. In fact, if the ln(1/(1 − x)) vs time plot is plotted (see Figure S3), it can be observed that the reaction did not follow first order kinetics with respect to styrene monomer, most likely due to a non-constant concentration of radicals. After 6 h of reaction 80% conversion was obtained for both pS30 and pSt50. The reaction was stopped at 6 h even if full conversion was not reached, since a living system was desired for the extension of the initial block with the soft monomer.

**Figure 3:**
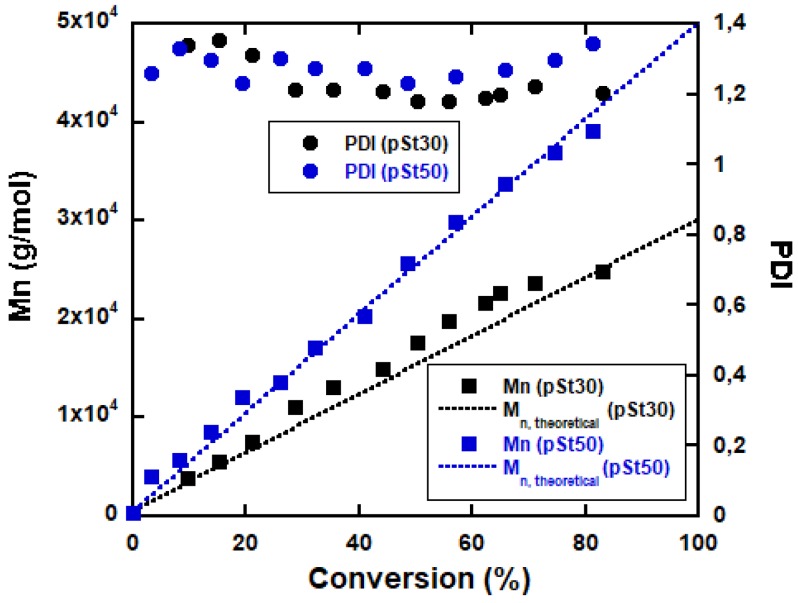
Plots of the number-average molecular weight and polydispersity index vs. monomer conversion for pSt30 and pSt50.

*M*_n_ for both pSt30 and pSt50 increased with conversion (Figure 3) following the theoretical prediction showing that the polymerization proceeded in well controlled manner. Moreover, the whole MWD shifted to higher molecular weight by increasing the conversion (see Figure S4a,b). Therefore, it could be considered that the polymerization was living and well controlled.

### 3.2. ABA Triblock Copolymer Latex

Once the initial pSt blocks were formed with 30,000 and 50,000 g/mol of targeted molecular weight (pSt30 and pSt50), the extension with the second soft monomer proceeded. The 2EHA monomer was fed as a pre-emulsion for three hours with additional amount of initiator added. Four block copolymers were synthesized having different molecular weights, named through the text as: p(St25-2EHA50-St25) as an extension of pSt50 and p(St15-2EHA50-St15), p(St15-2EHA70-St15) and p(St15-2EHA100-St15) as extensions of pSt30. The results of initial and the final block copolymers are shown in Table 1. The numbers after the letters indicate the targeted molecular weight. The second monomer was fed without any removal of the unreacted monomer (styrene) from the first step. Koiry and Singha studied the copper mediated controlled radical copolymerization of styrene and 2-ethylhexyl acrylate and determined their reactivity ratios to be r_1_ (styrene) = 1.29 and r_2_ (2EHA) = 0.73 [26]. Considering these reactivity ratios, the middle soft block is expected not to be pure poly(2EHA), but rather a gradient polymer that will still incorporate some styrene at the beginning of the polymerization of the second block. However, for simplicity reasons, this middle gradient block will be named as soft poly(2EHA) block throughout the text. Regarding the particle size, it can be seen that the particle size increased from the pSt seed to the block copolymers, indicating the growing of the particles by the fed 2EHA.

**Figure 4:**
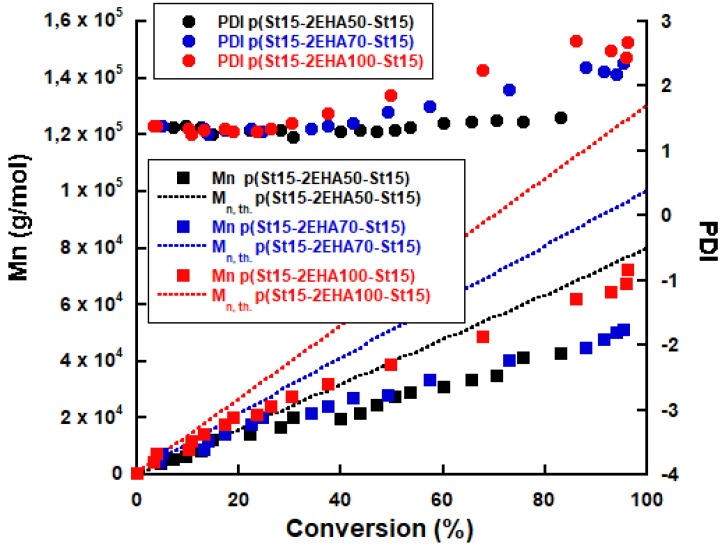
Evolution of the number average molecular weight and polydispersity index vs monomer conversion for different p(St-EHA-St) compositions.

The evolution of the *M*_n_ versus total monomer conversion (determined by solids content measurements) for block copolymers with different *M*_n_ prepared from pSt30 seed is shown in Figure 4. *M*_n_ grows linearly with conversion, however there is a visible negative deviation after the addition of the second monomer. The deviation gets more pronounced with the increase of the polymerized middle soft block. This is most likely due to the fact that the *M*_n_’s obtained from GPC were based on pSt standards and the Mark-Houwink constant of p2EHA was not taken into consideration. Equation (2) presents how the molecular weights of two different polymers can be related using the Mark-Houwink equation. In the equation K_1_, α_1_ and K_2,_ α_2_ represent the Mark-Houwink parameters for polymer 1 and 2, respectively, and M_1_ and M_2_ are the molecular weight of the polymers. Thus, as seen from Equation (2)—taking the constants for pSt (K = 0.000158 and α= 0.704) and p2EHA (K = 0.000124 and α= 0.667) into consideration—we were underestimating the real *M*_n_.

K_1_M_1_^(1+α_1_)^ = K_2_M_2_^(1+α_2_)^ (2)

**Figure 5:**
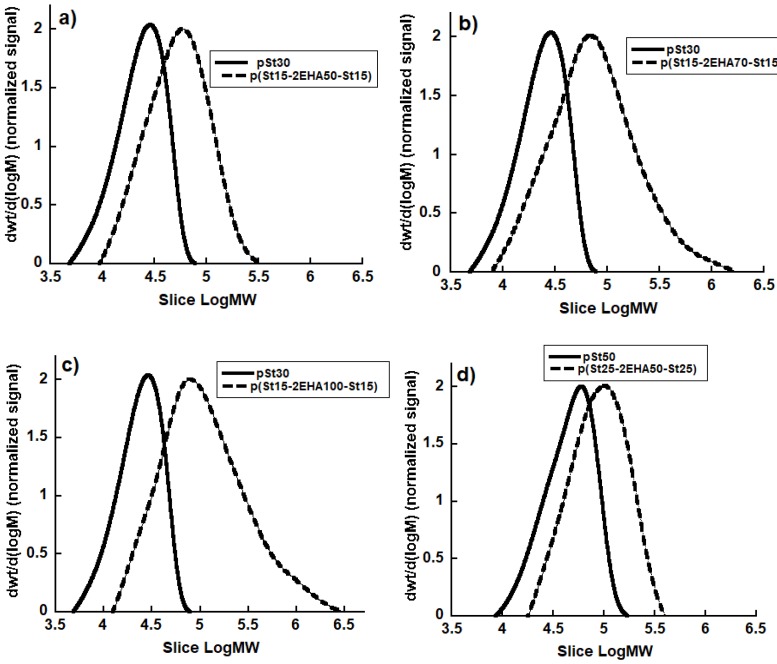
MWD of the initial pSt homopolymer (solid line) and final block copolymers (dashed line) of different pSt-EHA-St compositions.

Furthermore, it can be seen (Table 1) that all the final block copolymers have an overall conversion above 80% and the polydispersity index increased as the length of middle block was increased. Nevertheless, it should be noted that triblock copolymers were successfully formed, as is evident from the GPC curves (Figure 5), where the MWD of the final block copolymer moved to higher molecular weights compared to initial pSt block.

Widening of the MWD in the region of high molecular weight was observed when a higher molecular weight was targeted (for p(St15-2EHA70-St15) and p(St15-2EHA100-St15) Figure 5b,c respectively). This is most likely due to branching reactions, characteristic for acrylates [27,28] occurring as a result of combination of intermolecular chain transfer to polymer, which leads to long chain branches in the polymer, and termination by combination of branched growing radicals, leading to network formation and eventually gel polymer.

### 3.3. Thermal Properties of the Initial Homopolymers and Final Block Copolymers

The glass transition temperatures of the pSt homopolymers and p(St-2EHA-pSt) block copolymers were determined by DSC analysis from the samples dried at room temperature (Table 2).

Polystyrene initial blocks having different molecular weights showed a single *T*_g_ with very similar values (58 °C for pSt30 and 54 °C for pSt50). The *T*_g_ values obtained are much lower than the *T*_g_ of pSt reported in the literature (100–107 °C) [29]. This comes from the fact that the reactive co-stabilizer stearyl acrylate was used to prevent Oswald ripening in the miniemulsion. When stearyl acrylate is polymerized, a semi-crystalline polymer is obtained with a low *T*_g_ of −100 °C and a melting temperature of 50 °C [30]. Thus, when styrene copolymerizes with stearyl acrylate, its *T*_g_ is reduced. Another possible reason for the low *T*_g_ is the fact that the initial pSt blocks are of low molecular weight. Fox and Flory [31] studied the dependence of second-order transition temperature of polystyrene on the molecular weight and concluded that the *T*_g_ for high molecular weight polystyrene is 100 °C and decreases linearly with 1/*M*_n_ [31]. Therefore, both the addition of reactive co-stabilizer SA in the miniemulsion and the relatively low molecular weight pSt block can explain the *T*_g_s around 54–58 °C that have been found for the first block.

The triblock copolymers on the other hand exhibited two distinct transition temperatures—a lower and an upper one (as seen from Figure S5 in the Supplementary Material). The lower transition temperature ranges from −43 to −60 °C, depending on the composition of the block copolymers, and it is associated with the soft middle block. The difference in the values obtained for the lower *T*_g_ is due to the difference in the composition of the soft middle block. The block copolymers with higher overall composition of pSt contain soft middle block chains that bear higher amount of St units (unreacted from the first step). As a result, the *T*_g_ of the soft domains shifts to higher temperatures closer to the *T*_g_ of the pSt. On the contrary, block copolymers with higher overall amounts of p2EHA are composed of middle soft chains bearing more 2EHA units. Thus, the lower *T*_g_ of this block copolymer will get closer to the *T*_g_ of p2EHA (−60 °C) [26]. The upper transition temperature ranges from 55 to 63 °C and corresponds to the *T*_g_ of the pSt initial block. Therefore, there is a clear indication of a two-phase system.

Furthermore, the thermal stability of the copolymers was studied by TGA in nitrogen atmosphere and the results are summarized in Table 3 (see also Figure S6 in the Supplementary Material). The temperature at 10% weight loss was taken as *T*_onset_. *T*_max_ represents the maximum degradation temperature at which polymer back-bone starts degrading and forming a lot of volatile decomposition products and was determined from the TGS thermograms at the maximum of the derivative curve. It can be seen from the results that as the styrene content in the block copolymers increased, there was a slight increase in *T*_onset_. *T*_max_ on the other hand decreased as the length of the middle block increased. Moreover, DBTTC degradation started at 240 °C, and at 300 °C it was completely degraded. All the block copolymers showed a single step decomposition at rather high temperatures well above 200 °C, which is comparable to similar block ABA acrylic block compolymers synthesized by RAFT miniemulsion polymerization [22].

### 3.4. Morphology of the Block Copolymers

Block copolymer morphology was studied both by TEM and AFM. Due to the complexity of the system, i.e., mixed monomer sequence arising from the presence of unreacted monomer (initial block: styrene + stearyl acrylate and middle block: styrene + 2-ethylhexylacrylate), it was not possible to exactly determine the interaction parameter χ and also not possible to precisely determine the volume fraction of each microphase. Thus, it was not possible to precisely predict the equilibrium morphology of the block copolymers. However, considering the publication by Lee et al. [32] and Wang and Robertson [33], an interaction parameter of 0.025 for polystyrene and p(2EHA) can be estimated. Considering the smallest block copolymer p(St15-2EHA50-St15), its N was around 450 (156 (pSt + pSA) + 294 p2EHA). Therefore, χN was 11.25, which is above the phase separation limit (10.5). This is an indication that phase separation for all block copolymers can be expected. An estimate of the volume fraction of each component in the block copolymers was made based on the compositional information derived from NMR (Figure S1 in the Supplementary Material). The assumption that the weight fraction of monomers is equal or close enough to the volume fraction was made and the results are shown in Table 4. As stated in the review article of Mai and Eisenberg [34], the morphology of AB block copolymers changes when increasing the volume fraction f_A_ of the A block at a fixed χN > 10.5. When the volume fraction of a hard domain (such as polystyrene) is small (<20 vol %) spheres of polystyrene dispersed in an elastic matrix are formed, which then change to cylinders or gyroids as the pSt content increases. When the volume fractions of both components are about equal (40–60 vol %), the two-component form alternating lamellae. The prediction of the theoretical morphologies of the synthesized block copolymers was made based on the volume fractions calculated from NMR and the morphology diagram by Bates at al. [35], see Table 4.

**Figure 6:**
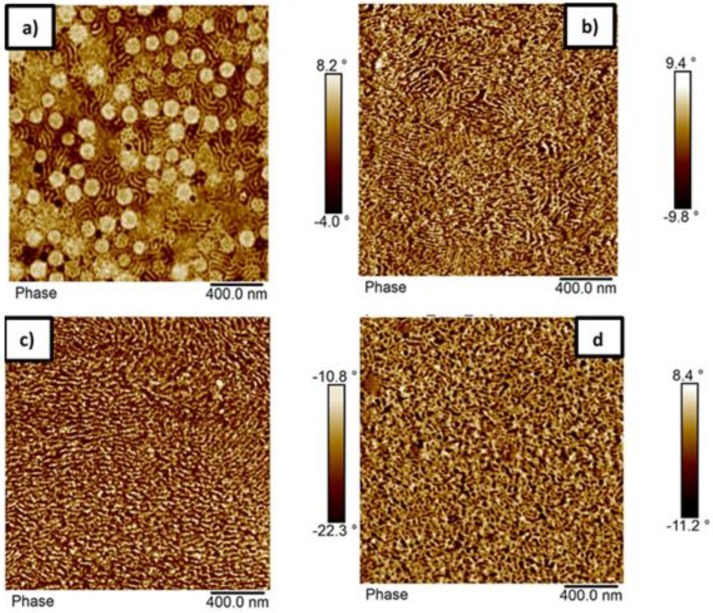
AFM phase images of the sample p(St15-2EHA50-St15): (**a**) top surface of the film dried at room temperature; (**b**) cross-section of the film dried at room temperature; (**c**) cross-section of the film dried at 100 °C, and; (**d**) cross section of a film cast from THF-solution.

The morphology of the block copolymer with composition p(St15-2EHA50-St15) was analyzed by AFM and the results are shown in Figure 6. The top surface of the polymer film prepared by drop casting of the latex at room temperature (Figure 6a) reveals the presence of spherical particles with hard shell (shown as bright regions), which are smaller than the particle size obtained by DLS (187 nm), dispersed in a nanophase separated continuous matrix, which most likely formed as a result of particle coalescence. The cross section of the AFM latex film dried at room temperature, (Figure 6b) showed no presence of particles—instead, an extended nanophase separated pattern was visible. The most likely reason for observing a regular structure already at room temperature is the fact that this sample contained a substantial amount of unreacted monomer (as seen in Table 1), acting as a plasticizer and causing particles to coalesce already at room temperature. On the other hand, the sample annealed at 100 °C (Figure 6c) showed even higher ordering compared to the sample obtained at room temperature. Nevertheless, no significant difference in morphology was seen between the films annealed at higher temperature and the ones cast from THF solution, indicating that equilibrium morphology was already reached by thermal treatment of the films. According to the diagram for AB block copolymers, the sample p(St15-2EHA50-St15), based on its volume fraction, should phase separate either in cylinder or gyroid structure depending on the interaction parameters. If we take into account Figure 6b–d, it seems that the gyroid morphology is the predominant one for this block copolymer.

**Figure 7:**
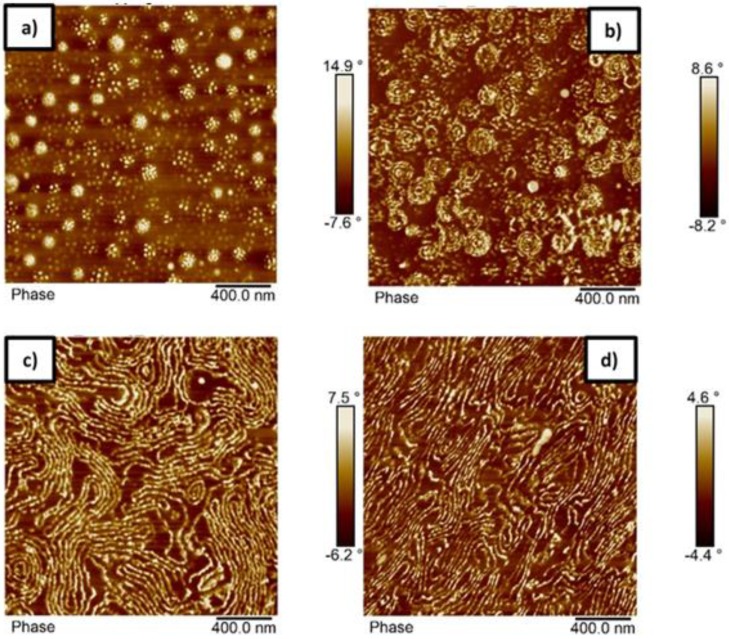
AFM phase images of the sample p(St15-2EHA70-St15) (**a**) top surface of the film dried at room temperature (**b**) cross-section of the film dried at room temperature, (**c**) cross-section of the film dried at 100 °C and (**d**) cross-section of a film cast from THF-solution.

**Figure 8:**
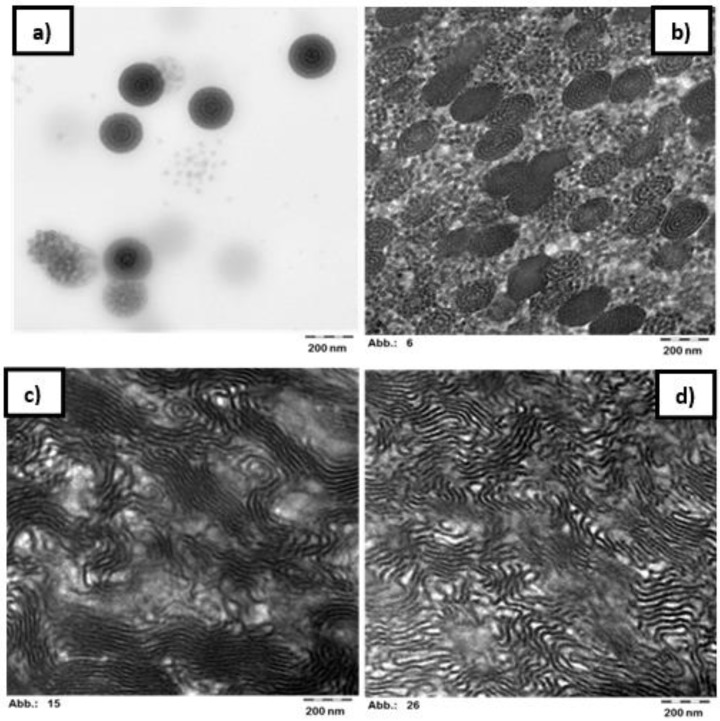
TEM images of the sample p(St15-2EHA70-St15) stained with RuO_4_ (**a**) particles dispersion embedded in hydroxyethyl cellulose (**b**) film dried at room temperature (**c**) film annealed at 100 °C and (**d**) film cast from THF-solution. The bar of all the images is 200 nm.

The self-assembly of the block copolymer with composition p(St15-2EHA70-St15) was studied by AFM (Figure 7) and compared to TEM images (Figure 8). The top surface of the polymer film (Figure 7a) studied by AFM shows the existence of two different populations of particles having lower and higher amount of styrene—visible as white dots. The AFM imaging performed on a cross-section of a latex film dried at room temperature reveals the presence of spherical particles inside of which “onion-ring” lamellar morphology can be distinguished (Figure 7b).

These findings were also confirmed by TEM (Figure 8b) where it is clearly visible that almost all the particles show the same structure. In the TEM images, the styrene rich phase appears dark due to the RuO_4_ staining. Moreover, from the TEM images (Figure 8a,b) it is seen that the pSt rings are perforated, which could be the reason for observing the white dots in the particles in AFM (Figure 7a). Although measurements derived from AFM are only an approximation, the size of the spherical objects shown in Figure 7b is in good agreement with the average particle size measured by DLS (189 nm). The thermal treatment of the polymer film caused complete particle coalescence and transformed the “onion-ring” structure into more classical lamellar morphology. To completely erase the impact of the emulsion polymerization process and thermal history, the latex film was dissolved in THF (Figure 7d). This yielded a morphology as close as possible to equilibrium morphology and the obtained structure resembled the one obtained at 100 °C, although the presence of onion-ring structures was not completely erased in the 100 °C annealed film, as it was after the THF treatment.

Based on the general diagram, block copolymers with a volume fraction of hard domain in the range of 20% should phase separate as either spheres or cylinders. On the contrary, the microscopic data clearly demonstrates that p(St15-2EHA70-St15) block copolymer phase separates into lamellar structure. However, as Matsen and Thompson stated in their article [36], ABA block copolymers phase separate slightly different than AB block copolymers. They predicted that the lamellar region for ABA block copolymers was reached for lower f_A_ compared to AB block copolymers, which is exactly what has been observed for p(St15-2EHA70-St15) in this study.

**Figure 9:**
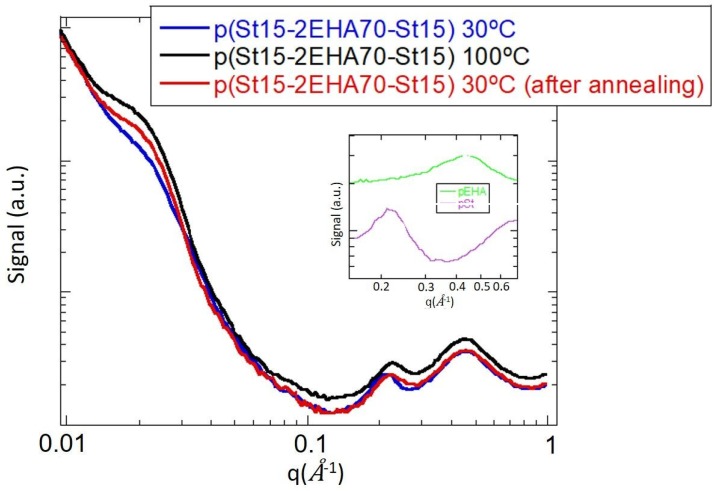
SAXS of the sample p(St15-2EHA70-St15) dried at room temperature and measured at 30, 100 and 30 °C after the analysis at 100 °C. Inserted: SAXS of pSt and pEHA homopolymers obtained by RAFT polymerization.

In order to confirm the lamellar structure, SAXS analysis of p(St15-2EHA70-St15) sample was carried out (Figure 9).

As can be seen, the spectra present three main bands at 0.02, 0.2 and 0.5 Å^−1^. The last two bands have been attributed to pSt (0.2 Å^−1^ and p2EHA 0.5 Å^−1^), but the one at 0.209 Å^−1^ can be attributed to the interlamellar distance between soft and hard phases. This q would mean an average interlayer distance of 30 nm. The interlamellar spacing is less developed in the sample dried at room temperature, but it is more clearly seen when the sample is annealed at 100 °C (maximum phase separation between soft and hard phases). The interlayer distance of 30 nm fits with the interlayer distances that can be measured in TEM images (Figure 8c).

**Figure 10:**
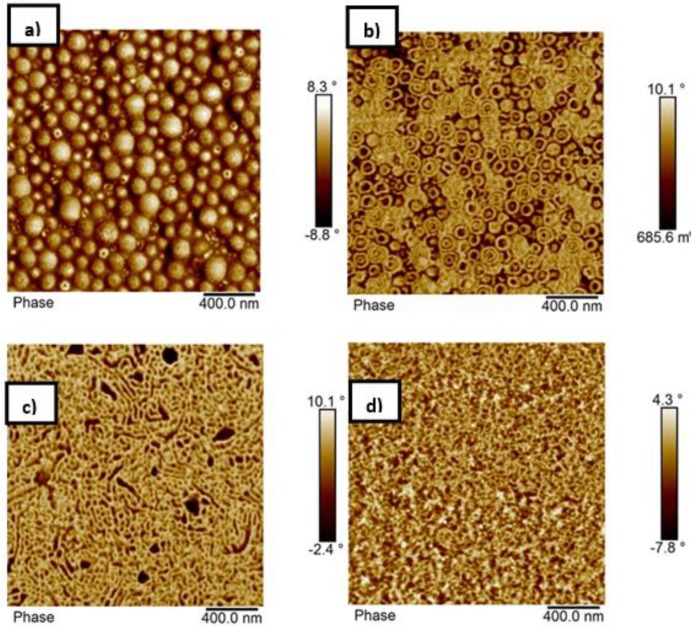
AFM phase images of the sample p(St25-2EHA50-St25): (**a**) top surface of the film dried at room temperature; (**b**) cross-section of the film dried at room temperature; (**c**) cross-section of the film dried at 100 °C and; (**d**) cross section of a film cast from THF solution.

The sample p(St15-2EHA100-St15) with the longest soft block was very sticky, thus it was not possible to analyze by microscopy. The sample with highest pSt content p(St25-2EHA50-St25) showed hard spherical particles with no particular outer morphology as evident form the AFM image of the top surface of the polymer film (Figure 10a). AFM images of the cross section of the film dried at room temperature on the other hand showed single or bilayer particle morphology (Figure 10b). At this stage, the differences observed for the top and cross-sections of the films dried at room temperature have to be pointed out. In all three latexes analyzed so far, the differences have been significant, but they are very clear here. The top view only shows the surface of the particles, whereas the cross-section shows their interior. If only the top view would have been considered, no phase separation would have been envisaged from Figure 10a. On the other hand, annealing of the film led to complete coalescence of the hard particles and homogeneous distribution of the p2EHA domains through the hard pSt matrix (Figure 10c). Presence of small voids was also visible in annealed films, which could not be seen in the film obtained from THF solution (Figure 10d). According to the general diagram based on the volume fraction either lamellar or gyroid structure should be expected for this sample. The images obtained, especially from the film cast from THF-solution 10d, suggest that we had a gyroid structure.

### 3.5. Viscoelastic Properties of the ABA Hard-Soft-Hard Block Copolymers

**Figure 11:**
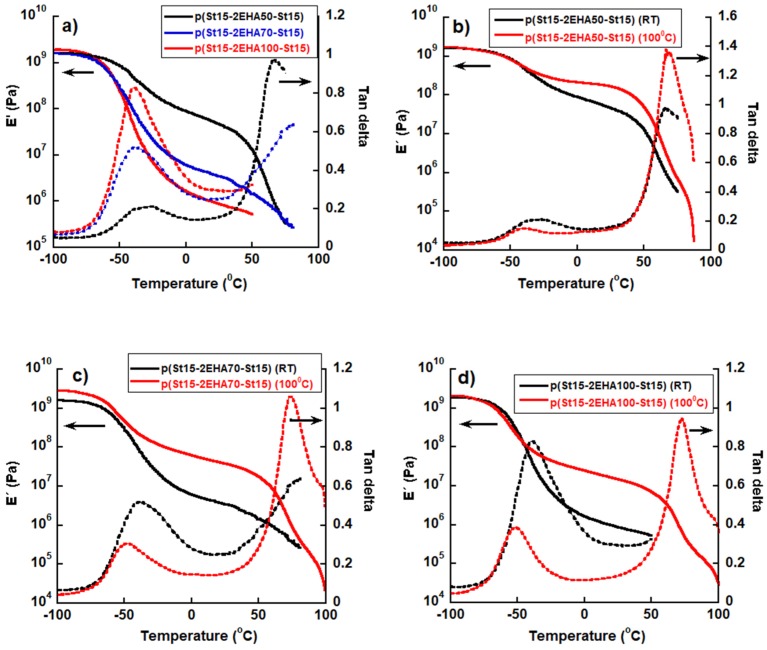
Viscoelastic properties of the block copolymers. Solid lines: dependence of elastic modulus on temperature. Dashed lines: dependence of tan δ on temperature.

To investigate whether the obtained morphological changes observed during annealing influenced the viscoelastic properties of films, the hard-soft-hard block copolymers were investigated by DMTA and the results are presented in Figure 11.

The solid line presents the influence of block copolymer composition or annealing temperature on storage modulus and the dashed line the effect on tan δ. Viscoelastic properties of the films cast at room temperature presented in Figure 11a indicate that the elastic modulus decreased as the length of the middle soft p2EHA block increased. The elastic modulus decreased significantly in the rubbery plateau, especially in the temperature range between 0 and 50 °C. If the AFM images of Figure 6 (p(St15-2EHA50-St15)) and Figure 7 (p(St15-2EHA70-St15)) are observed, it is clear that the periodic size of the 2EHA domains is much smaller for the first case (around 400 nm for p(St15-2EHA50-St15) block copolymer) than for the second one (more than 1 µm for (p(St15-2EHA70-St15)). Therefore, the higher modulus of p(St15-2EHA50-St15) can be attributed to smaller soft 2EHA domains. Moreover, liquid-like behavior induced by further temperature increase was evident from the significant drop of the modulus and abrupt increase in tan δ at temperatures close to the *T*_g_ of the hard domains. The viscoelastic properties of p(St25-2EHA50-St25) were not possible to be measured, because the sample was too brittle and handling was very difficult.

Additionally, in Figure 11b–d the viscoelastic properties of the block copolymers latex films cast at room temperature were compared with the ones of the latex films annealed at 100 °C. Shifting the *T*_g_ (of soft domains) to lower temperatures, an elastic modulus increase in the plateau region was clearly visible in all the samples upon annealing, irrespective of the composition of the block copolymers. When films were annealed, complete particle coalescence occurred as evidenced from the AFM images, thus pSt domains were able to move and uniformly distribute throughout the elastic matrix, which led to its reinforcement. As a result, hard thermoplastic rubber-like materials were obtained with increased elastic modulus in the plateau region. When the films were dried at room temperature the contact between blocks was most likely higher, and thus their influence on each other was higher. As a result, the lower and upper *T*_g_’s approached each other. On the other hand, when they were annealed, less contact was achieved and the influence of the blocks on each other was lower. As a result, the two *T*_g_’s got apart from each other. In addition, the upper *T*_g_ of the hard domain got more pronounced, most likely due to the fact that the domains got bigger by annealing and the interface region between the microdomains in which segments of both blocks mix got narrower.

## 4. Conclusions

Waterborne ABA block copolymers of hard A block and soft B block were synthesized via two-step reversible addition fragmentation chain transfer polymerization, using *S,S*-dibenzyl trithiocarbonate bifunctional (DBTTC) RAFT agent. First, min-iemulsion polymerization was used for the synthesis of the hard-polystyrene domains where the reaction proceeded up to 80% conversion in a controlled manner. Then, in the second step, 2EHA was fed to the system as a pre-emulsion and soft domains were formed containing a small fraction of styrene from the initial step. The successful formation of ABA block copolymer was proven by MWD shift and linear increase in *M*_n_. However, it was also observed that there was a negative deviation of the *M*_n_ obtained versus the theoretical *M*_n_, which most likely originated from the fact that the *M*_n_ of the block copolymers was determined from the GPC running with pSt standards only.

Furthermore, the thermal properties of both the homopolymers and the block copolymers were investigated by DSC and it was shown that the initial PSt homopolymers showed unexpectedly low *T*_g_ because stearyl acrylate was used as a co-stabilizer due to the low *M*_n_ of the blocks. The block copolymers showed the presence of two *T*_g_s corresponding to the soft and hard domains in the system. Good thermal stability and a single step decomposition was observed for the block copolymers studied by TGA. The effect of particle morphology on the film morphology at different thermal treatments for various types of block copolymers was investigated by means of microscopic techniques. It was shown that when the films were cast at room temperature, the deformation and coalescence of the particle was not complete and their nanophase separation was preserved. Once thermal treatment was applied, particle coalescence and ordering on a bigger length scale was achieved, which was close to the equilibrium morphology. Furthermore, it was shown that the composition of the block copolymers, as well as the thermal treatments of the polymer films greatly influenced the viscoelastic properties of the block copolymers. Finally, it has to be underlined that after annealing, hard thermoplastic rubber-like materials were obtained with increased elastic modulus in the plateau region. This opens the door to the use of these materials in the field of thermo-responsive materials.

## Figures and Tables

**Table 1:** Conversion, average droplet (dd) and particle size (dp), *M*_n_ and PDI at the end of reaction for pSt homopolymers and p(St-2EHA-St) block copolymers.

Reactions	Conversion (%)	*M*_n,GPC_ (g/mol)	PDI	dd (nm)	dp (nm)	Conversion (%)	dp (nm)	*M*_n,GPC_ (g/mol)	PDI
	pSt Homopolymers					p(St-2EHA-St) Block Copolymers			
p(St25-2EHA50-St25)	81.2	39,119	1.34	119	159	84.0	177	73,854	1.45
p(St15-2EHA50-St15)	81.8	20,204	1.22	116	154	83.1	187	42,877	1.52
p(St15-2EHA70-St15)	82.0	20,044	1.29	130	161	95.2	187	51,046	2.36
p(St15-2EHA100-St15)	82.0	20,044	1.29	130	161	96.3	207	72,203	2.6

**Table 2:** Thermal properties of the pSt and p(St-2EHA-St) block copolymers obtained by DSC.

Material Code	*T*_g1_ (°C)	*T*_g2_ (°C)
DSC analysis of the samples dried at 23 °C		
pSt30	58	-
pSt50	54	-
p(St25-2EHA50-St25)	−43	58
p(St15-2EHA50-St15)	−58	55
p(St15-2EHA70-St15)	−60	60
p(St15-2EHA100- St15)	−60	63

**Table 3:** Thermal stability of the block copolymers measured by TGA.

Material Code	T_onset_ (°C)	*T*_max_ (°C)
TGA analysis performed in nitrogen atmosphere		
DBTTC	239.9	288.4
p(St25-2EHA50-St25)	372.7	413.8
p(St15-2EHA50-St15)	365.2	402.7
p(St15-2EHA70-St15)	364.0	397.7
p(St15-2EHA100-St15)	361.4	396.6

**Table 4:** Block copolymer composition results determined by ^1^H NMR spectroscopy and theoretical morphology predicted based on AB block copolymers model.

Sample	Mole Fraction		Weight Fraction		Theoretical Morphology [34]
	% St	%2EHA+SA	%St+SA	%2EHA	
p(St25-2EHA50-St25)	61.0	39.0	40.6	59.4	L/G
p(St15-2EHA50-St15)	55.3	44.7	35.7	64.3	G/C
p(St15-2EHA70-St15)	35.6	64.4	20.8	79.2	S/C
p(St15-2EHA100-St15)	33.2	66.8	19.2	80.8	S

S—Spheres; G—Gyroid, C—Cylinders; L—Lamellae.

## Supplementary Materials

The following are available online at https://www.mdpi.com/2073-4360/11/8/1259/s1, Figure S1: 1HNMR spectrum of a representative p(St-EHA-St) block copolymer. Figure S2. Stability of the St miniemulsion containing 2% Dowfax 2A1 and 1% of Disponil A3065 BOM at 60 °C. Figure S3. Conversion (x) and ln(1/(1-x)) plots for the RAFT miniemulsion polymerization of pSt30 and pSt50. Figure S4. Molecular weight distribution of A) pSt30 and b) pSt50 obtained by IR refractive index detector. Figure S5. DSC thermograms of the p(St-EHA-St) block copolymers measured at a heating rate of 10 °C/min. Figure S6. TGA thermograms of the p(St-EHA-St) block copolymers measured at 10 °C/min in nitrogen.
