# Supplementary material for: Mechanical and Morphological Properties of Waterborne ABA Hard-Soft-Hard Block Copolymers Synthesized by Means of RAFT Miniemulsion Polymerization
Source: Polymers (Basel). 2019 Jul 30;11(8):1259. doi: 10.3390/polym11081259 (PMC6722633; doi:10.3390/polym11081259)
Supplement: Supplementary file 1 [file polymers-11-01259-s001.pdf]

Supplementary Material

Mechanical and morphological properties of waterborne ABA hard-soft-hard block copolymers synthesized by means of RAFT miniemulsion polymerization

Gordana Siljanovska Petreska [a,b], Arantxa Arbe[c], Clemens Auschra[b] and Maria Paulis[a]*

[a]POLYMAT, University of the Basque Country UPV/EHU, Joxe Mari Korta Center, Avda. Tolosa 72, 20018 Donostia-San Sebastián, Spain

[b]BASF SE, 67056 Ludwigshafen – Germany

[c]Centro de Física de Materiales (CFM) (CSIC--UPV/EHU) -- Materials Physics Center (MPC), Paseo Manuel de Lardizabal 5, 20018 Donostia-San Sebastián, Spain

For the TEM measurements, the block copolymers dispersions having hard domains were embedded in Natrosol HR 250 = hydroxyethylcellulose (HEC) and stained with RuO_4. The latex films were prepared by casting the latex into silicone molds and drying them for a week at room temperature. Annealed films were obtained by casting the latex and drying them at room temperature for 48 h and then annealing the film at 100°C for 96 h. Solvent cast films on the other hand were prepared by redissolution of the films in tetrahydrofuran followed by casting and drying at room temperature. Ultrathin cross sections (about 100 nm thick, perpendicular to the surface) of RuO_4 stained films were prepared via cryo ultramicrotomy (Leica UC 7). The sections were examined with a Zeiss Libra 120 transmission electron microscope with an omega filter operating at an accelerating voltage of 120 kV in elastic mode.

AFM imaging was done using Bruker Dimension Icon AFM using Olympus OMCL-AC160TS cantilever for tapping with a resonant frequency of 300 kHz and spring constant of 42N/m (34-50). Films surface and films cross sections, cryocut at −80 °C, were analyzed by AFM.

SAXS experiments were conducted on a Rigaku 3-pinhole PSAXS-L equipment operating at 45 kV and 0.88 mA. CuK_α transition photons of wavelength $\lambda = 1.54$ Å are produced by a MicroMax-002+ X-Ray Generator System that is composed by a microfocus sealed tube source module and an integrated X-Ray generator unit. Flight path and sample chamber in the equipment are under vacuum. A two-dimensional multiwire X-Ray Detector (Gabriel design, 2D-200X) detects the scattered X-Rays. This gas-filled proportional type detector offers a 200 mm diameter active area with ca. 200 micron resolution. Samples were placed in a Linkam Scientific Instruments THMS 600 temperature controller (range: -196 to 600ºC, stability < 0.1ºC) in transmission geometry

with sample to detector distances of 2 m and 50 cm. The azimuthally averaged scattered intensities were obtained as a function of wavevector q, $q = 4\pi\lambda^{-1} \times \sin(\theta)$, where 2θ is the scattering angle. Combining these configurations, the q-range covered was $0.008 \leq q \leq 1.0$ Å^{-1}. The latex film dried at room temperature was inserted in the equipment, and SAXS analysis was carried out at 30ºC, at 100ºC and at 30ºC again after cooling the sample. Reference pSt and pEHA homopolymers obtained by RAFT polymerization were also analyzed.

The composition of the block copolymers was determined from the NMR results using the following equation:

$$F_{EHA} = \frac{5A}{5A+2B} x100$$

where, F_{EHA} is the fraction of 2EHA in the block copolymer, A the integral for -OCH_2- protons in p2EHA (at δ=3.7) and B represents the integral for aromatic protons of polystyrene unit (at δ=6.5-7.3 ppm) (see Figure S3). It was assumed that the conversion of stearyl acrylate monomer was complete.

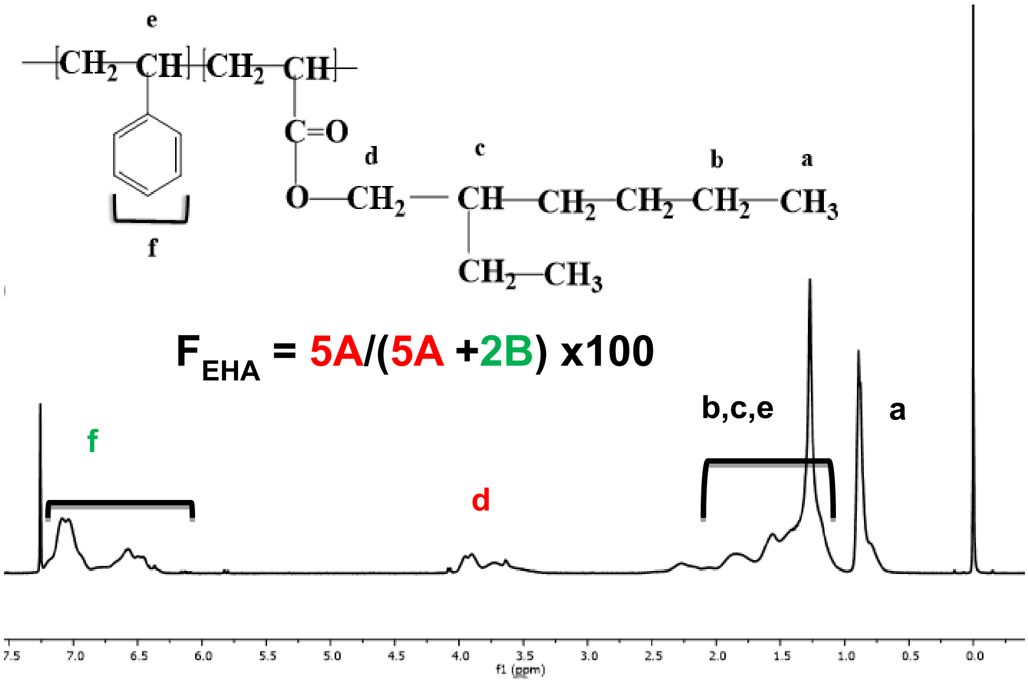

Figure S1. 1HNMR spectrum of a representative p(St-EHA-St) block copolymer.

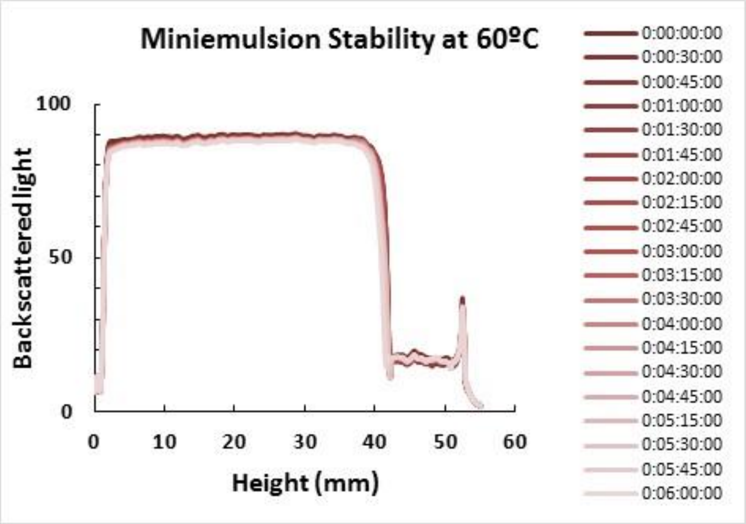

Figure S2. Stability of the St miniemulsion containing 2% Dowfax 2A1 and 1% of Disponil A3065 BOM at 60 °C.

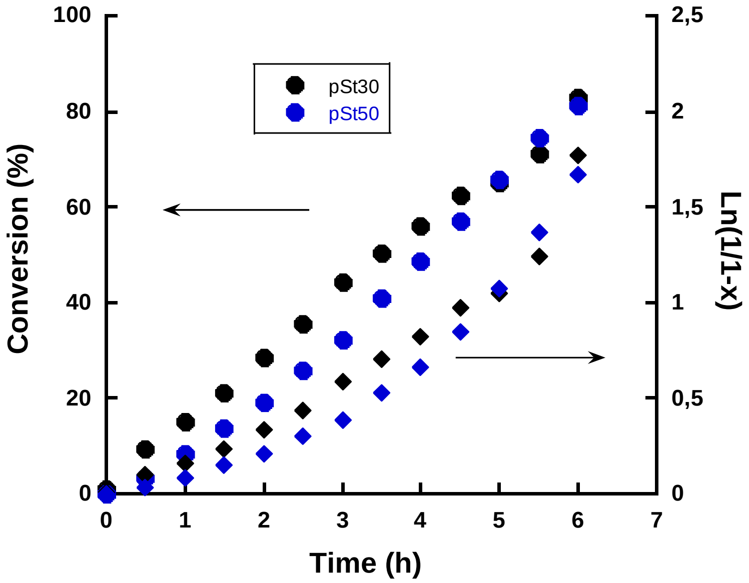

Figure S3. Conversion (x) and ln(1/(1-x)) plots for the RAFT miniemulsion polymerization of pSt30 and pSt50.

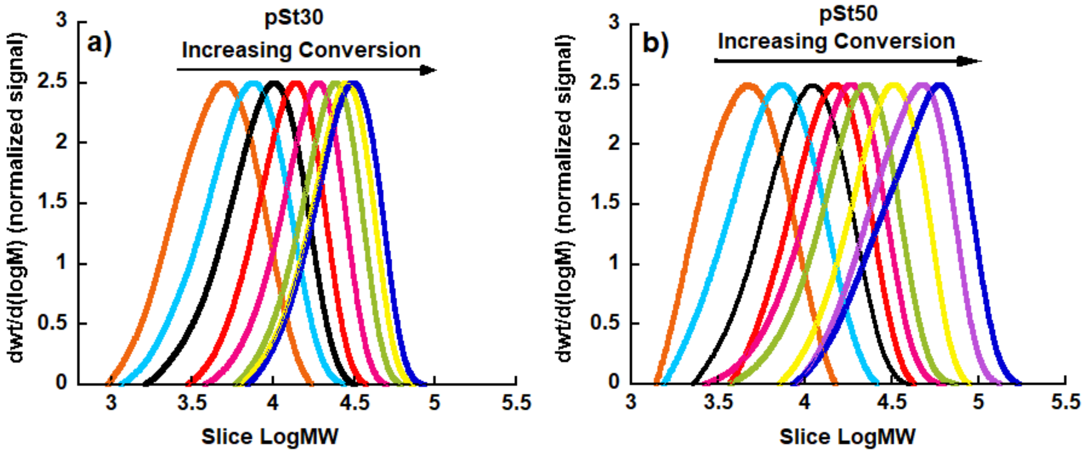

Figure S4. Molecular weight distribution of A) pSt30 and b) pSt50 obtained by IR refractive index detector.

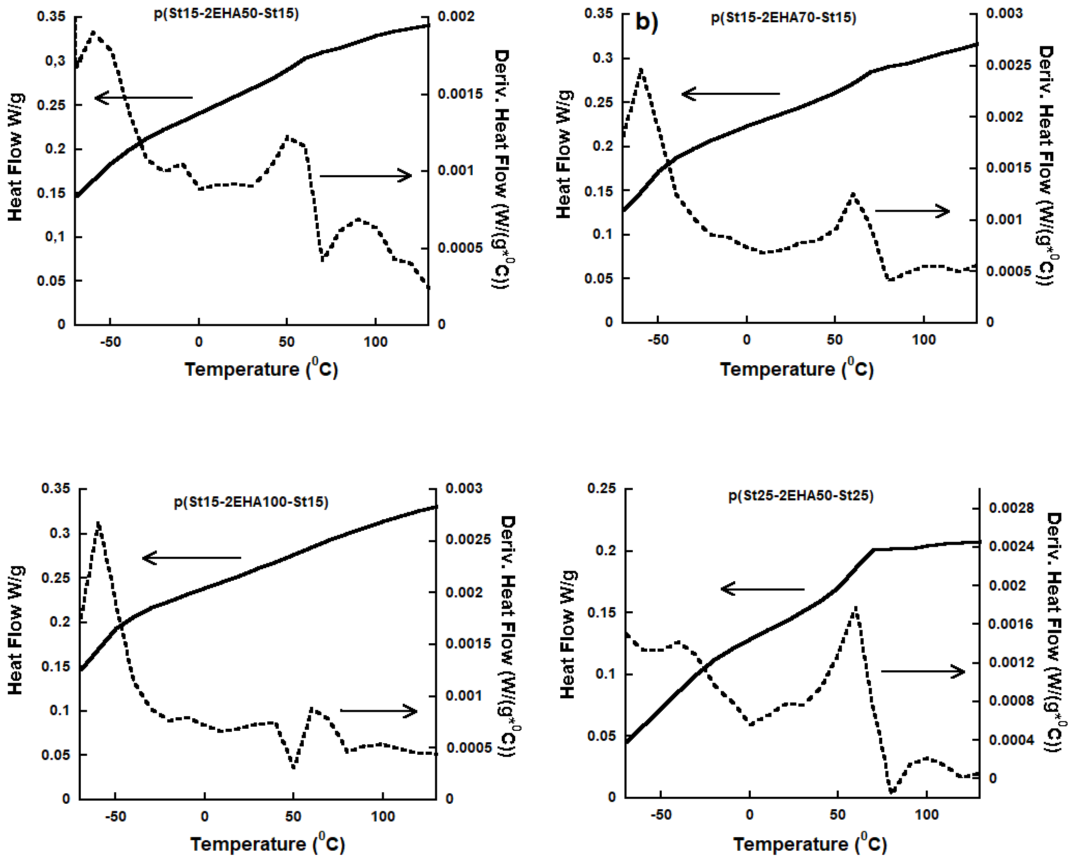

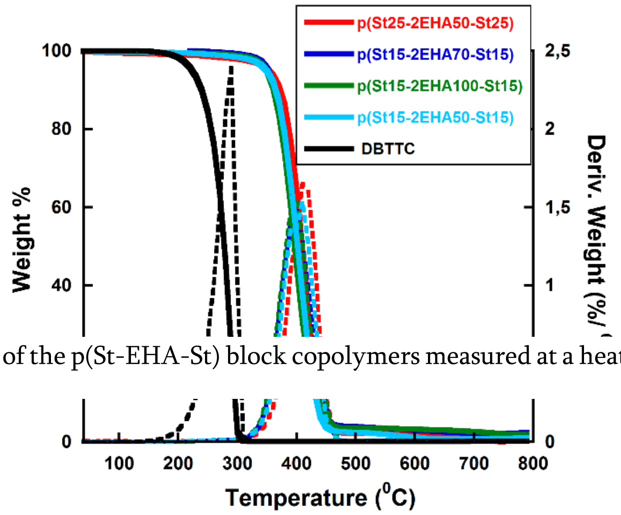

Figure S5. DSC thermograms of the p(St-EHA-St) block copolymers measured at a heating rate of 10 °C/min.

Figure S6. TGA thermograms of the p(St-EHA-St) block copolymers measured at 10 °C/min in nitrogen.
